# Endometrial Cancer Suppresses CD8+ T Cell-Mediated Cytotoxicity in Postmenopausal Women

**DOI:** 10.3389/fimmu.2021.657326

**Published:** 2021-04-23

**Authors:** Mickey V. Patel, Zheng Shen, Marta Rodriguez-Garcia, Edward J. Usherwood, Laura J. Tafe, Charles R. Wira

**Affiliations:** ^1^ Department of Microbiology and Immunology, Geisel School of Medicine at Dartmouth, Lebanon, NH, United States; ^2^ Department of Immunology, Tufts University School of Medicine, Boston, MA, United States; ^3^ Department of Pathology, Dartmouth-Hitchcock Medical Center, Lebanon, NH, United States

**Keywords:** endometrial cancer, cytotoxicity, CD8+ T cells, granzyme, perforin

## Abstract

Endometrial cancer is the most common gynecological cancer. To investigate how it suppresses host immune function, we isolated CD8+ T cells from endometrial endometroid carcinomas and adjacent non-cancerous endometrium and determined if the tumor environment regulates cytotoxic capacity. Endometrial carcinomas had increased numbers of CD8+ T cells compared to adjacent non-cancerous endometrium. Tumor CD8+ T cells expressed significantly less granzyme A (GZA), B (GZB), and PD-1 than those in adjacent non-cancerous tissues and also had significantly lower cytotoxic killing of allogeneic target cells. CD103-CD8+ T cells, but not CD103+CD8+ T cells, from both adjacent and tumor tissue were primarily responsible for killing of allogeneic target cells. Secretions recovered from endometrial carcinoma tissues suppressed CD8+ cytotoxic killing and lowered perforin, GZB and PD-1 expression relative to non-tumor CD8+ T cells. Furthermore, tumor secretions contained significantly higher levels of immunosuppressive cytokines including TGFβ than non-tumor tissues. Thus, the tumor microenvironment suppresses cytotoxic killing by CD8+ T cells *via* the secretion of immunosuppressive cytokines leading to decreased expression of intracellular cytolytic molecules. These studies demonstrate the complexity of CD8+ T cell regulation within the endometrial tumor microenvironment and provide a foundation of information essential for the development of therapeutic strategies for gynecological cancers.

## Introduction

Endometrial cancer is the most common gynecological cancer worldwide and the sixth most common cause of cancer death in women (1–4). It occurs primarily in postmenopausal women with an average age of diagnosis of 60 years, and in recent years is characterized by both a rising incidence and mortality rate (1–4). Thus, endometrial cancers represent a significant health burden to postmenopausal women. Endometrial cancers encompass a broad range of malignancies and are subdivided into type I (endometrioid carcinoma) and type II (serous, clear cell, carcinosarcoma, dedifferentiated and mixed types). Of these, endometrioid carcinomas account for 80% of total endometrial carcinomas (5). Despite its increasing prevalence, little is known about the immunology of endometrial carcinomas, particularly the role of immune cells in tumor control.

Previous studies have shown that cytotoxic CD8+ T cells are essential for tumor control, and their presence is linked to positive outcomes in multiple cancers (6, 7). CD8+ T cells are crucial inhibitors of tumor growth with their ability to kill tumor cells *via* the action of perforin, granzymes and other cytokines (8). In endometrial carcinomas the presence of CD8+ tumor infiltrating lymphocytes (TILs) is associated with a better prognosis (9). The contribution of CD8+ T cells to tumor control varies between tissue-resident (CD103+) and non-resident (CD103-) cells, with CD103+ cells associated with enhanced antitumor immunity (10). The presence of high numbers of CD103+CD8+ TILs is also associated with improved outcomes in endometrial carcinomas (11). How the tumor microenvironment controls the function of CD8+T cells remains unclear but CD8+ T cells represent a potent level of immune protection whose contribution to control of endometrial cancer is unknown.

CD8+ T cells in the noncancerous endometrium are a unique population, distinct from those in blood, whose cytotoxic capacity varies with stage of life (12–15). Using a redirected cell lysis assay, we demonstrated that cytotoxic killing of allogeneic target cells by endometrial CD8+ T cells varies with stage of the menstrual cycle, reaching its nadir during the secretory phase and peak during proliferative phase (16). Cytotoxic killing by CD8+T cells was significantly higher in postmenopausal women compared to premenopausal women irrespective of menstrual cycle stage (16). This presents a contradictory observation: since most endometrial cancers are diagnosed in postmenopausal women, tumor growth occurs when the cytotoxic capacity of endometrial CD8+T cells should be at their highest. This suggests that tumors compromise the cytotoxic capacity of endometrial CD8+T cells. More recently, in addition to confirming these findings using a direct cell lysis assay, we found that endometrial CD8+ T cell cytotoxic killing varies with the co-expression of CD103, the marker for tissue residence, with tissue-resident endometrial CD103+CD8+ T cell cytotoxic killing suppressed compared to non-resident CD103-CD8+ T cells (17). Whether there is a differential contribution to tumor control between CD103+ versus CD103- CD8+ T cells in endometrial cancer remains unclear.

Recognizing the potential importance of CD8+ T cells in restricting endometrial tumor growth, we investigated the regulation and cytotoxic capacity of CD8+ T cells from matched non-cancerous versus endometrial carcinoma tissue. We show that CD8+ T cell cytotoxicity is suppressed in endometrial carcinomas compared to adjacent non-cancerous tissue. Furthermore, CD103-CD8+ T cells have greater cytotoxic capacity compared to CD103+CD8+ T cells in both adjacent and tumor endometrium. Additionally, granzyme A (GZA) and B (GZB), and PD-1 expression is decreased in CD103-CD8+ T cells from endometrial carcinomas compared to adjacent non-cancerous tissue. Finally, endometrial tumor secretions, containing a spectrum of immunosuppressive molecules including TGFβ, that contribute to the suppression of cytotoxic killing by CD8+ T cells in cancerous tissue.

## Methods and Materials

### Patient Population

Endometrial carcinomas, the majority of which were endometrioid type, and adjacent non-cancerous endometrial tissue were obtained from women following hysterectomy at Dartmouth-Hitchcock Medical Center (Lebanon, NH) under the guidance of a pathologist (LJT). Tumors were consecutively collected with the primary selection criteria being the availability of 1 cm^2^ of uninvolved endometrium. Patient ages ranged from 45-86 years old, with a median age of 64.5 years (**Table 1**). Studies were approved by the Dartmouth College Institutional Review Board and the Committee for the Protection of Human Subjects (CPHS) with written informed consent obtained prior to surgery.

**Table 1:** Endometrial cancer patient characteristics.

**Number of Patients**	40
**Median Age (*years*)**	64
**Age Range (*years*)**	45-86
**FIGO Grade (*n*)**	
** 1**	*21*
** 2**	*11*
** 3**	*8*
**FIGO Stage (*n*)**	
** IA**	*28*
** IB**	*8*
** IIIA**	*1*
** IIIC1**	*3*
**pT status (*n*)**	
** 1a**	*31*
** 1b**	*9*
**pN status (*n*)**	
** 0**	*35*
** 1a**	*2*
** 1mi**	*1*
** X**	*2*
**MMR status (*n*)**	
** Intact**	*30*
**MLH1 & PMS2 loss**	*7*
**MSH2 & MSH6 loss**	*1*
** PMS2 loss**	*1*
** MSH6 loss**	*1*

### Tissue Processing

Endometrial tumor tissue and adjacent non-cancerous tissues were processed under sterile conditions following surgery. Our established enzymatic digestion protocol has been adopted to minimize breakdown of cell surface markers and has been extensively validated by us in multiple studies across multiple cell types (17–23). Briefly, tissues were rinsed with 1X Hanks Buffered Saline Solution, cut into 1-2mm pieces, and digested for 45 min at 37°C in an enzyme mix containing 0.05% collagenase type IV (Sigma-Aldrich, St. Louis, MO) and 0.01% DNAse (Worthington Biochemical, Lakewood, NJ). Following incubation, digested tissues were passed through a 250µm mesh filter (Small Parts, Miami Lakes, FL) to remove any remaining large tissue fragments. The flow-through was sequentially filtered through 40µm and 20µm filters to remove epithelial sheets and tumor cells, and the resulting flow-through containing the remaining mixed stromal cells and immune cells recovered for further processing as described below.

### CD103- and CD103+ CD8+ T Cell Isolation

CD8+ T cells were purified from the mixed cell suspension using a negative magnetic bead selection kit (Miltenyi Biotec, Auburn, CA) as previously described by us (17, 23). Dead cells were removed using the Dead cell removal kit (Miltenyi Biotec). To remove stromal fibroblasts from the mixed cell suspension anti-fibroblasts microbeads were also added. Purity of the CD8+ T cell population was higher than 90% following two rounds of negative selection (17, 23).

To separate CD103-CD8+ T cells from CD103+CD8+ T cells, the purified CD8+ T cell suspension was incubated with CD103-PE antibody (Miltenyi Biotec) for 10 minutes after which anti-PE ultra-pure magnetic beads (Miltenyi Biotec) were added. CD103- were separated by negative selection and CD103+ by positive selection resulting in a purity range between 90-95% for CD103- and CD103+ CD8+ T cells respectively (17, 23).

### Flow Cytometry

Cell surface markers from the mixed cell suspensions were stained with various combinations of antibodies: CD45-vioblue 450, CD8-FITC (Tonbo), CD3-viogreen (Miltenyi), CD45-AF700, CD3-APC-Cy7, CD4-APC-Cy7, CD103-BV711 (BioLegend), CD4-PE-Cy5.5, CD103-PE-Cy7 (eBioscience, San Diego, CA), CD8-BUV395 (BD Bioscience). Cell surface marker expression was measured by the percentage of positive cells analyzed using BioRad ZE5 flow cytometers (BioRad) with Everest software. Data was analyzed with FlowJo Software v.10 (Tree Star Inc., Ashland, OR, www.flowjo.com). The authors acknowledge the Immune Monitoring and Flow Cytometry Resource at the Norris Cotton Cancer Center at Dartmouth with NCI Cancer Center Support Grant (5P30 CA023108-41) and COBRE Grant (P30GM103415-15) from the National Institute of General Medical Sciences.

### Intracellular Staining

Detection of perforin (PRF), granzyme A (GZA) and B (GZB) was performed on mixed cell populations after dead cell removal. Cells were surface stained to identify CD103- and CD103+ CD8+ T cells and then fixed and permeabilized with Cytofix/cytoperm kit (BD) according to manufacturer’s instructions. Intracellular staining of perforin, GZA and GZB were performed using combinations of the following antibodies: anti-human Perforin-PE/Dazzle, Granzyme A-AF647, Granzyme A-PerCp-Cy5.5, Granzyme B-AF647 (all BioLegend) and Granzyme B-BV421 (BD Bioscience).

### Preparation of Blood CD4+T Cells for Cytotoxicity Assay

Blood Leuko Paks for cytotoxicity experiments were obtained from the DHMC Blood Donor Program. Blood donors were anonymous, no information regarding age or hormonal status was available, and only female donors were used in this study. Peripheral blood mononuclear cells (PBMCs) were recovered by standard Ficoll density gradient centrifugation. Blood CD4+T cells were isolated from PBMCs by negative magnetic bead selection using a CD4+T cell isolation kit (Miltenyi Biotec). Following isolation, CD4+T cells were stored in liquid N_2_ until use. Once thawed, the CD4+T cells were resuspended in X-Vivo 15 media (Lonza, Walkersville, MD) supplemented with 10% charcoal stripped human AB serum (Valley Biomedical, Winchester, VA) prior to cytotoxicity assays (17, 23).

### Preparation of Blood CD8+T Cells for Cytotoxicity Assay

PBMCs were recovered by standard Ficoll density gradient centrifugation from blood Leuko-Paks obtained from the DHMC Blood Donor Program. Blood CD8+T cells were isolated from PBMCs by negative magnetic bead selection using a CD8+T cell isolation kit (Miltenyi Biotec). Following isolation, CD8+ T cells were stored in liquid N_2_ until use. Once thawed, CD8+T cells were resuspended in X-Vivo 15 media with IL-2 (50 U/ml, AIDS Research and Reference Reagent Program, Division of AIDS, NIAID, NIH: Human rIL-2 from Dr. Maurice Gately, Hoffmann- La Roche Inc.) for 24 hrs prior to cytotoxicity assays. Female donors for blood CD8+T cells were different from blood CD4+T cell donors.

### Cytotoxicity Assay

To determine cytotoxic killing by CD8+ T cells from endometrial tumors and non-cancerous adjacent tissue, we performed cytotoxicity assays as previously described (17, 23). Allogeneic blood CD4+ T cells were stained with CFSE (Cell Division Tracker Kit; BioLegend) and then co-cultured with purified total CD8+ T cells (or CD103+CD8+ or CD103-CD8+ as indicated) in 96-well plates at an effector to target ratio of 1:1. Cytotox Red (Essen Biosciences) was added to the CD4+/CD8+ culture to stain dead cells. Using the IncuCyte Zoom system (Essen Biosciences), plates were imaged every 10 minutes and dead CD4+ target cells identified as double green (CFSE) and red (Cytotox) stained cells. Controls included CD4+T cells alone, CD8+T cells alone, X-Vivo 15 media alone, and Cytotox Red alone. Cytotoxicity was calculated by determining the average number of dead CD4+T cells over the first 4 hrs as previously described (17, 23).

To determine the effect of conditioned media (See below) on cytotoxic killing by blood CD8+T cells, conditioned media was diluted by 50% in fresh X-Vivo 15 media with IL-2 (50 U/ml) and incubated with blood CD8+T cells isolated from healthy female donors for 24 hrs. Following incubation, the conditioned media was washed out using X-Vivo 15 media and blood CD8+T cells mixed with allogeneic blood CD4+T cells at an effector to target ratio of 1:1. Cytotoxic killing was measured as described above.

### Conditioned Media

Mixed cell conditioned media was generated by incubating fresh stromal suspensions at 1x10^6^ cells/ml from either adjacent non-cancerous tissue or endometrial carcinoma tissue in X-Vivo 15 media for 24 hrs at 37°C. After 24 hrs, media was recovered and centrifuged at 800 x g to remove any cellular debris and the supernatant aliquoted prior to being stored at -80°C until used.

### Measurement of Protein Secretion in Conditioned Media

A 42-plex Luminex (EMD Millipore, Burlington, MA) and TGFβ ELISA assays (R&D Systems, Minneapolis, MN) were used to characterize the profile of soluble mediators produced by mixed cell suspensions of endometrial carcinoma or adjacent non-cancerous tissues according to the manufacturers’ instructions using a Bio-Plex Array Reader (Bio-Rad, Hercules, CA) (Luminex) or Epoch Microplate Spectrophotometer (BioTek, Winooski, VT) (TGFβ).

### Statistics

Data analysis was performed using GraphPad Prism version 8.3 software for Windows (GraphPad Software, www.graphpad.com). A two-sided *P*-value <0.05 was considered statistically significant. Comparison of two groups was performed with the non-parametric test U–Mann Whitney or Wilcoxon paired test. Comparison of three or more groups was performed applying the non-parametric Kruskal-Wallis test followed by Dunns post-test.

## Results

### Endometrial Carcinomas Are Enriched for Immune Cells

To define the functional capacity of CD8+ T cells in endometrial cancer, we received matched tissue samples from endometrial carcinomas and adjacent non-cancerous endometrial tissue from patients undergoing surgery at DHMC. Thirty-six tumors were endometrioid type, one serous, one carcinosarcoma and two mixed. As seen in **Table 1**, our patient population (*n=40*) had a median age of 64 years, with only one individual below 50 years. Overall, the majority of patients had low-grade, early stage tumors (FIGO grade1 (53%) or 2 (28%), and FIGO stage IA (70%) or IB (20%)).

**Figure 1:**
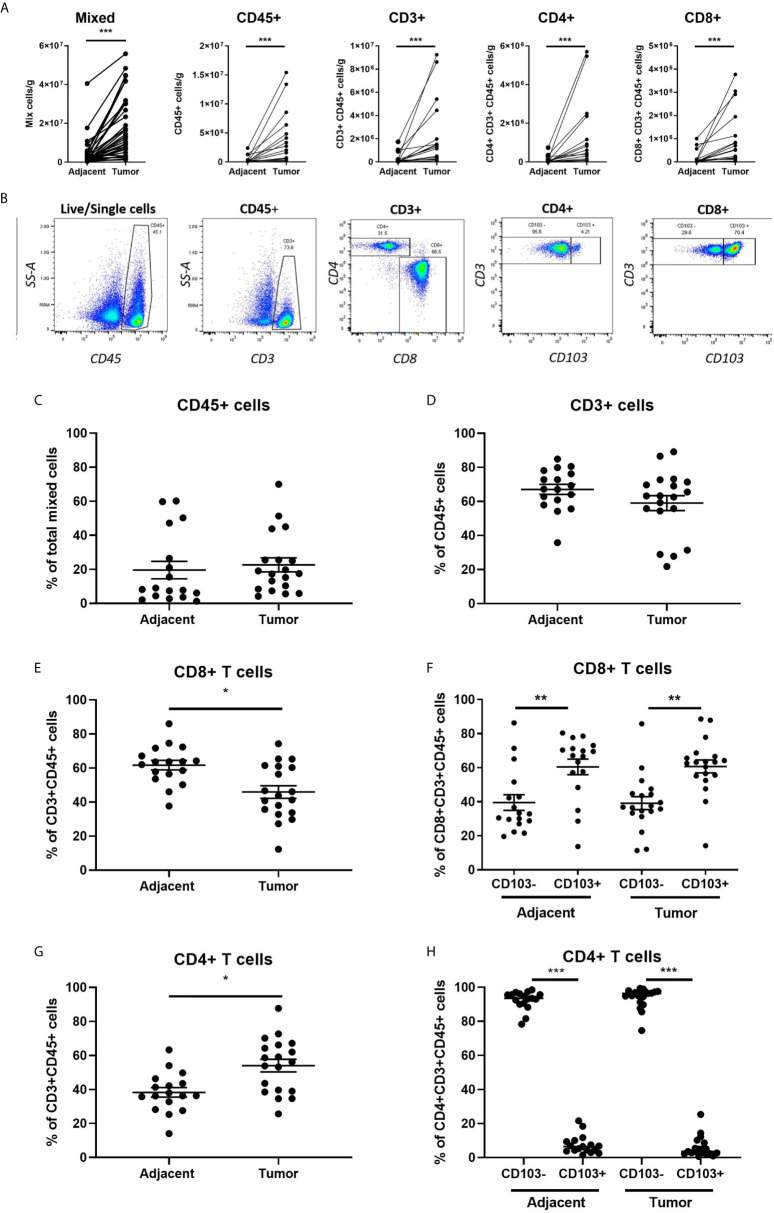
Increased Immune Cell Numbers in Matched Endometrial Carcinomas versus Adjacent Non-Cancerous Tissues. The number of cells per gram of tissue in different subsets (total mixed cells, CD45+, CD45+CD3+, CD3+CD4+, and CD3+CD8+) from matched endometrial carcinomas and adjacent non-cancerous tissue **(A)**. Representative flow cytometry gating panel to determine the presence of CD45+, CD3+, CD4+, CD8+, and CD103+ cells in mixed cell preparations from both adjacent non-cancerous endometrium and endometrial carcinoma tissues **(B)**. The percentage of total CD45+ **(C)**, CD3+ **(D)**, CD8+ **(E)**, CD4+ **(F)** T cells, and CD103 +/- CD8+ **(G)** and CD4+ **(H)** T cells in matched endometrial carcinoma and adjacent non-cancerous tissue was determined by flow cytometry. Each circle represents an individual patient. *p<0.05, **p<0.01, ***p<0.001. Wilcoxon matched-pairs signed rank test.

We initially determined the immune cell composition of matched adjacent non-cancerous and endometrial carcinoma tissues. As seen in **Figures 1A–B**, endometrial carcinomas have a significantly greater number of total mixed, CD45+, CD3+, CD4+CD3+, and CD8+CD3+ cells per gram of tissue compared to adjacent non-cancerous tissue. Cell numbers within tumor or adjacent tissue did not vary significantly with FIGO grade or stage (data not shown). This demonstrates that endometrial carcinomas are enriched with immune cells, including CD8+ T cells, compared to non-cancerous endometrium.

Despite an overall increase in the number of CD45+ and CD3+ cells in endometrial carcinomas, as a percentage of total mixed cells (**Figure 1C**) and CD45+ cells (**Figure 1D**), their respective proportions did not change between tumor and adjacent tissue. However, while the total number of CD8+ T cells increased in endometrial carcinomas (**Figure 1A**), they significantly decreased as a percentage of CD3+ cells compared to adjacent non-cancerous tissue (43% versus 63%) (**Figure 1E**). In contrast, the percentage of CD4+ T cells increased in endometrial carcinomas (**Figure 1G**). We then characterized the proportion of CD8+ T cells that were tissue-resident (CD103+) versus non-resident (CD103-). As seen in **Figure 1F**, in both non-cancerous and tumor endometrial tissue, the percentage of CD103+CD8+ T cells was significantly higher than the percentage of CD103-CD8+ T cells (adjacent: CD103- 33% versus CD103+ 67%) (tumor: CD103- 37% versus CD103+ 63%). This demonstrates that the majority of CD8+ T cells in both tumor and non-cancerous endometrial tissue are tissue-resident (CD103+) cells. In contrast, there were fewer CD103+CD4+ T cells from both tumor and non-cancerous and tissues than CD103-CD4+ T cells, comprising less than 10% of the total population of CD4+ T cells at both sites (**Figure 1H**).

### CD8+ T Cell Cytotoxic Killing Is Suppressed in Endometrial Carcinomas

**Figure 2:**
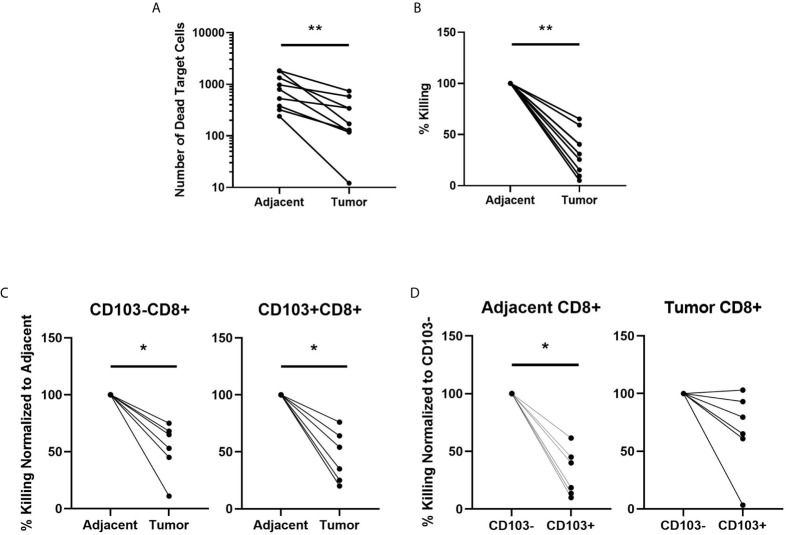
CD8+ T cell Cytotoxic Killing is Suppressed in Endometrial Carcinomas Compared to Adjacent Non-Cancerous Tissue. The capacity of total CD8+ T cell cytotoxic killing of allogeneic target cells was determined using an IncuCyte Zoom and expressed as the difference in number of dead target cells **(A)** and the percentage dead cells in adjacent non-cancerous tissue **(B)** over 4hrs. The value of killing in each endometrial carcinoma is expressed as percentage of its matched adjacent non-cancerous tissue which is set to 100% **(B)**. CD8+ T cells were separated into CD103- or CD103+ subpopulations from both the adjacent non-cancerous endometrium or endometrial carcinoma and cytotoxic capacity determined by IncuCyte Zoom comparing adjacent CD103- versus tumor CD103-CD8+ T cells and adjacent CD103+ versus tumor CD103+CD8+ T cells **(C)** over 4 hrs. Cytotoxic killing of allogeneic target cells by tumor CD103+ and CD103- CD8+T cells is normalized to % killing by adjacent CD103+ or 103- CD8+ T cells, which is set to 100%. Cytotoxic killing was also determined between adjacent CD103- versus adjacent CD103+, and tumor CD103- versus tumor CD103+ **(D)** cells over 4hrs. In D, cytotoxic killing of allogeneic target cells by CD103+CD8+ T cells is expressed as a percentage of killing by CD103-CD8+ T cells, which is set to 100%. Each circle represents an individual patient. *p<0.05, **p<0.01. Wilcoxon matched-pairs signed rank test.

CD8+ T cells are essential for cancer control *via* their cytotoxic capacity to kill tumor cells (24). What remains unclear is their cytotoxic potential in endometrial cancer. To determine whether the cytotoxic capacity of CD8+ T cells against allogeneic target cells differed between adjacent and cancerous endometrium, we purified CD8+ T cells from adjacent and tumor endometrial tissue by magnetic bead isolation, before measuring cytotoxic function using time-lapse imaging. As seen in **Figures 2A–B**, killing of allogeneic target cells by CD8+ T cells recovered from endometrial carcinomas was significantly suppressed by approximately 50% compared to CD8+ T cells recovered from adjacent non-cancerous tissue. This demonstrates that despite an overall increase in CD8+ T cells in endometrial carcinomas, their cytotoxic capacity is suppressed in the tumor environment.

### Cytotoxic Killing by CD103+CD8+ T Cells Is Suppressed in Both Endometrial Carcinoma and Adjacent Non-Cancerous Tissue

CD103 is a marker of tissue residence at mucosal sites. Since increased numbers of CD103+CD8+ T cells in endometrial cancer are linked to positive prognostic outcomes (11), we determined whether cytotoxic capacity varied between tissue-resident CD103+CD8+ T cells and non-resident CD103-CD8+ T cells. Building upon our studies in **Figures 2A–B**, we used magnetic bead isolation to further separate the CD8+ T cells into CD103- and CD103+ fractions. We compared cytotoxic killing between adjacent and tumor tissue for CD103- and CD103+ (**Figure 2C**) CD8+ T cells. In both populations, tumor CD8+T cells had significantly lower cytotoxic killing of allogeneic target cells than CD103- and CD103+CD8+ T cells from adjacent non-cancerous tissue. In adjacent non-cancerous endometrial tissues (**Figure 2D**), we found that cytotoxic killing by CD103+CD8+ T cells was significantly lower than that seen with CD103-CD8+ T cells.

### Endometrial Carcinoma Microenvironment Selectively Modulates CD8+ T Cell Expression of Granzyme A, B, and PD-1

**Figure 3:**
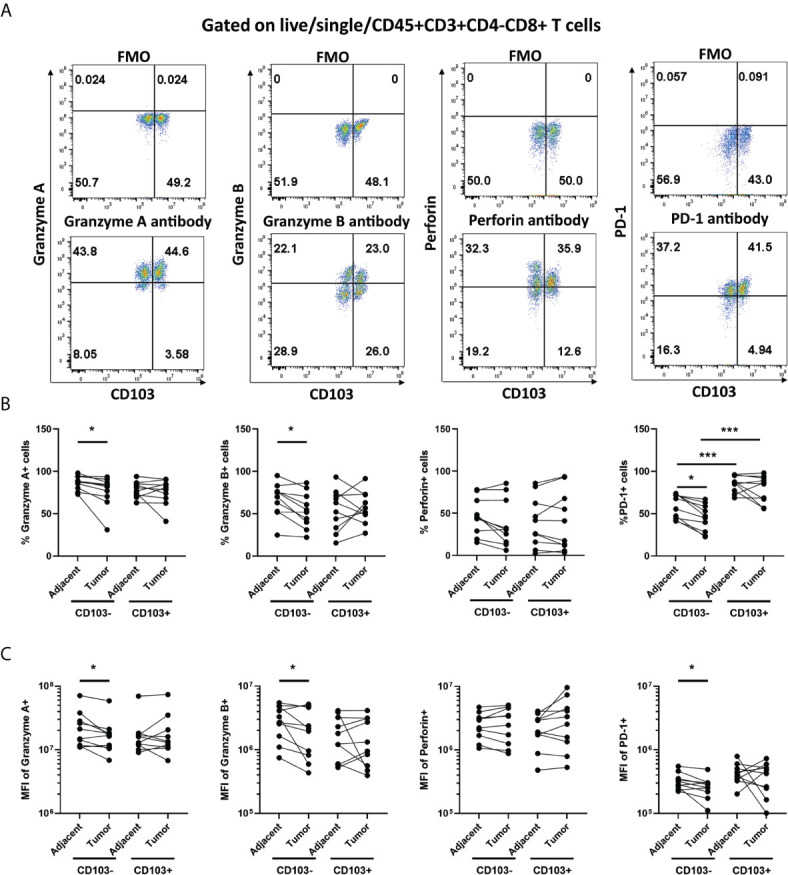
Granzyme A and B, and PD-1 Expression is Suppressed in Endometrial Carcinoma CD103-CD8+ T cells. The expression of Granzyme A, Granzyme B, Perforin, and PD-1 by CD103- and CD103+ CD8+ T cells from endometrial carcinomas and adjacent non-cancerous endometrium was determined by flow cytometry. Representative flow cytometry gating is indicated in panel **(A)**. Data is presented as percentage of positive cells **(B)** or mean fluorescence intensity (MFI) **(C)**. Each circle represents an individual patient. (*n=9*). *p<0.05; ***p<0.001. Wilcoxon matched-pairs signed rank test.

To understand the underlying mechanisms involved in the suppression of CD8+ T cell cytotoxic killing in endometrial tumors, we investigated whether there were changes in the expression of granzyme A (GZA), granzyme B (GZB), perforin (PRF), and PD-1 in CD103+ and CD103-CD8+ T cells. Expression of all 4 markers was determined as percent positive cells and mean fluorescence intensity (MFI) by flow cytometry. As seen in **Figures 3A–B**, there were significantly fewer GZA+, GZB+, and PD-1+ CD103-CD8+ T cells in endometrial tumors compared to adjacent non-cancerous endometrium. Similarly, MFI analysis showed that GZA, GZB, and PD-1 were significantly lower in tumor CD103-CD8+ T cells compared to non-cancerous CD103-CD8+ T cells (**Figure 3C**). No differences were observed in the percentage of PRF+ cells or PRF intracellular content between non-cancerous and tumor CD103-CD8+ T cells. In contrast to the CD103- population, there were no significant differences in either the percent of cells expressing GZA, GZB, PRF, and PD-1 or intracellular content measured in CD103+CD8+ T cells from non-cancerous or tumor endometrium. Overall, these findings indicate that CD103-CD8+ T cells in endometrial tumors, which are primarily responsible for cell killing (**Figure 2**), express lower levels of cytotoxic molecules than do their counterparts in adjacent non-cancerous tissues.

### Endometrial Carcinoma Secretions Suppress Blood CD8+ T Cell Cytotoxic Killing

Within the tumor environment cytotoxic suppression of CD8+ T cells can occur *via* contact-dependent and contact-independent mechanisms. To evaluate the contribution of secretions from endometrial carcinomas to the suppression of CD8+ T cell cytotoxicity we generated patient-matched mixed single cell suspensions of either non-cancerous or endometrial carcinoma tissues and collected conditioned media (CM) after 24 hrs incubation.

**Figure 4:**
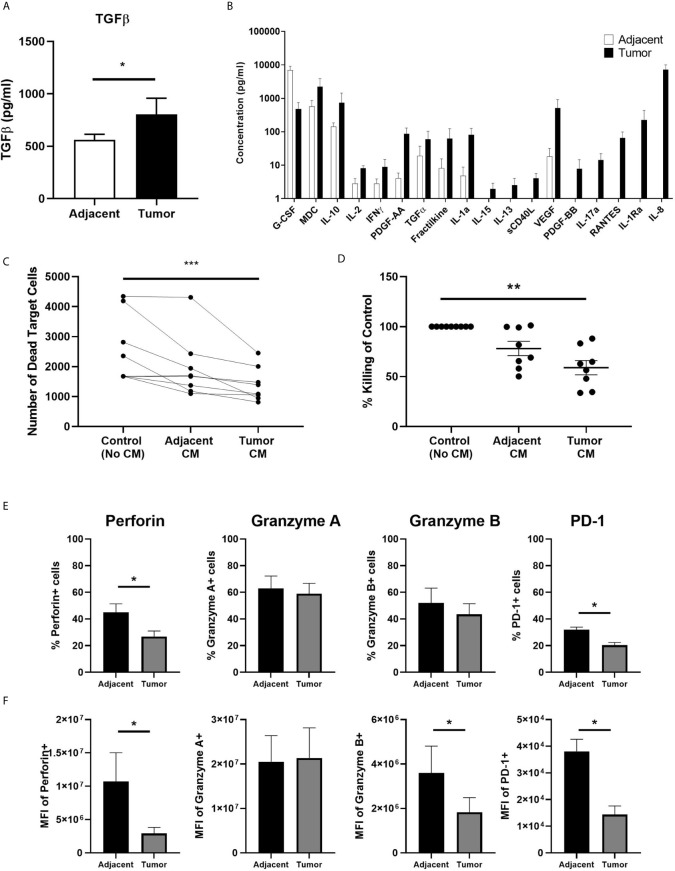
Secretions from Endometrial Carcinomas Suppress Blood CD8+ T cell Cytotoxic Killing. A 24 hr mixed cell conditioned media (CM) was generated from matched endometrial carcinoma and adjacent non-cancerous tissue. Secreted proteins in CM were measured using a TGFβ ELISA **(A)** and 41-plex Luminex **(B)**. Data is expressed as mean (pg/ml) +/- SEM secretion of adjacent non-cancerous tissue versus endometrial carcinoma. (Luminex: *n=4;* ELISA: *n=11*). Purified blood CD8+ T cells were preincubated with IL-2 and CM for 24 hrs after which cytotoxic killing capacity was determined. Data is presented as number of dead target cells **(C)** and percent cytotoxic killing compared to blood CD8+ T cells not incubated in CM whose value was set to 100% **(D)** with each circle representing an individual patient (*n=8*). CM from six matched endometrial carcinoma (Tumor) and adjacent non-cancerous (Adjacent) tissues was incubated with blood CD8+ T cells for 24 hrs prior to analysis of by flow cytometry of perforin, GZA, GZB, and PD-1 **(E, F)**. Data is expressed percentage of positive cells **(E)** or MFI **(F)** in blood CD8+ T cells +/- SEM. (*n=6*). *p<0.05, **p<0.01, ***p<0.001. Wilcoxon matched-pairs signed rank test **(A, B, E, F)** and non-parametric Kruskal-Wallis test followed by Dunn’s multiple comparisons test **(C, D)**.

Having demonstrated previously that TGFβ suppresses endometrial CD8+ T cell cytotoxic killing in non-cancerous patients (17), we measured the concentration of TGFβ by ELISA to determine whether tumor secretions contain higher concentrations of TGFβ than adjacent tissue. We found that tumor CM contained significantly higher levels of total TGFβ than adjacent CM (**Figure 4A**). Recognizing that CM likely contains a spectrum of soluble mediators that may suppress CD8+T cell cytotoxicity, we performed a Luminex assay (41-plex) to compare tumor and adjacent CM. As seen in **Figure 4B**, CM from adjacent non-cancerous endometrium and endometrial carcinoma contains a spectrum of molecules with broad immunological effects. The concentration of IP-10, PDGF-AA, TGFα, fractalkine, IL-1α, IL-15, IL-13, sCD40L, VEGF, PDGF-BB, IL-17, RANTES, IL-1Ra and IL-8 trended higher in CM from endometrial tumors than that measured in CM from adjacent non-cancerous tissue. In contrast, only G-CSF was higher in CM from non-cancerous tissue versus tumor CM. Thus, tumor CM contains a spectrum of cytokines capable of suppressing CD8+T cell cytotoxic killing.

To demonstrate whether tumor secretions were capable of inhibiting CD8+T cell cytotoxicity, we incubated freshly isolated blood CD8+ T cells for 24 hrs in CM prior to being mixed with allogeneic target cells. Our rationale for using blood CD8+T cells instead of tissue CD8+T cells was that our average recovery 1.8x10^5^ CD8+T cells/gm from adjacent tissue (**Figure 1**) was not sufficient to adequately perform the cytotoxicity assay. By using blood CD8+T cells from female donors instead of CD8+T cells from adjacent tissue we were able to perform the assay with sufficient controls and replicates. As seen in **Figures 4C–D**, CM from endometrial carcinomas significantly suppressed killing by blood CD8+ T cells by approximately 45% compared to blood CD8+ T cells alone. In contrast, CM from adjacent tissues trended towards suppression but were not significantly different from controls. This demonstrates that secretions from endometrial tumor tissue is capable of suppressing CD8+T cell cytotoxicity to a greater extent than secretions from non-cancerous endometrium in the same patient.

To determine whether CM from tumor tissues alters the expression of PRF, GZA, GZB, and PD-1, blood CD8+ T cells were incubated for 24 hrs in endometrial carcinoma CM prior to analysis by flow cytometry. As seen in **Figure 4E**, the number of PRF+ and PD-1+ CD8+ T cells was significantly reduced following incubation with tumor CM compared to adjacent CM. When intracellular content was measured, we found that PRF, GZB and PD-1 levels were reduced by 50-80% (**Figure 4F**). Tumor CM had no effect on the number of cells expressing, or the intracellular content of, GZA. These findings indicate that endometrial tumor secretions rapidly (within 24 hrs) suppress CD8+ T cell cytotoxic function leading to a reduction in their ability to kill target cells.

## Discussion

Our study demonstrates that endometrial adenocarcinomas contain increased total numbers of CD8+ T cells but significantly lower cytotoxic activity compared to adjacent non-cancerous endometrium. CD8+ T cells from endometrial adenocarcinomas express significantly less GZA, GZB, and PD-1 than CD8+ T cells compared to non-cancerous CD8+ T cells. Furthermore, CD103-CD8+ T cells from endometrial adenocarcinomas and adjacent non-cancerous tissues were primarily responsible for cytotoxic killing capacity. Lastly, endometrial adenocarcinoma secretions suppressed both cytotoxic killing by blood CD8+ T cells and intracellular expression of PRF, GZB and PD-1 to a significantly greater extent than secretions from non-cancerous tissue. Thus, endometrial adenocarcinomas are suppressive towards CD8+ T cell cytotoxic killing potentially creating an environment permissive for tumor growth and survival.

We report that endometrial carcinomas are enriched for immune cells with significantly greater numbers of both CD4+ and CD8+ T cells than that seen in adjacent non-cancerous endometrium. Increased numbers of CD4+ and CD8+ T cells could be due to the increased concentration of T cell chemokines such as RANTES present in endometrial carcinoma secretions (**Figure 4**) and may reflect the efforts of the host immune system to restrict tumor growth. Others have reported increased numbers of CD45+ cells and CD56+ NK cells within endometrial tumors (25). Similar to our results with CD8+ T cells, while the overall number of CD56+ NK cells increased, their frequency within the CD45+ immune cell population decreased in endometrial carcinomas (25). Together with our studies, this demonstrates that immune cell infiltrates composed of cytotoxic cells (CD8+ T cells and NK cells) are characteristic of endometrial carcinomas. The presence of high numbers of CD8+ T cells within endometrial carcinomas is associated with favorable prognostic factors and prolonged overall survival (9). Furthermore, the presence of CD8+ T cells is negatively correlated with histologic grade, myometrial invasion, and lymph node metastasis (26). This strongly suggests that CD8+ T cells represent a key avenue of tumor control (24).

To the best of our knowledge, we are the first to demonstrate that CD8+ T cell cytotoxic killing is significantly suppressed in endometrial carcinomas compared to adjacent non-cancerous endometrium. Furthermore, while CD103+ and CD103- CD8+ T cells are present in endometrial tumors, the majority of cytotoxic activity is due to non-resident CD103- CD8+ T cells which account for approximately 40% of the CD8+ T cells in the tumor. The lack of cytotoxic activity by CD103+CD8+ T cells presents a conundrum since they account for over 60% of total CD8+ T cells in the tumor and adjacent non-cancerous tissue. Paradoxically, the presence of high CD103+CD8+ T cell infiltration in endometrial carcinomas is associated with an improved prognosis (11). Resident CD103+CD8+ T cells are known to play a key role in suppressing tumor growth in other solid cancers. For example, in gastric cancer CD103+CD8+ T cells have greater anti-tumor effects, including increased cytotoxicity than CD103-CD8+ T cells (27). One avenue to reconcile these contradictory observations is that the improved prognosis associated with increased numbers of CD103+CD8+ T cells was most pronounced in high-risk endometrial cancer patients (11). This category was defined by the authors as FIGO stage II, III, and IV as well as all non-endometrioid cancers (11). In contrast, the majority of patients in our study were from FIGO stage I. Thus, CD103+CD8+ cytotoxic function may be greater in higher risk patients compared to the low risk patients in our population sample. Alternatively, CD103+CD8+ T cells may have a role in tumor suppression beyond direct cytotoxic killing through the secretion of TNFα (28) and IFNγ (29), or the recruitment of other immune cell populations that in turn may limit tumor growth. This underlies the importance for further studies to investigate how the cytotoxic function of CD8+ T cells varies in later stages of endometrial cancer.

The mechanistic basis for increased suppression of CD103+ versus CD103-CD8+ T cell cytotoxicity remains unclear. Our results are consistent with our previous findings that CD8+ T cells in the normal pre- and post-menopausal endometrium consist of both CD103+ and CD103- T cells, with the latter being primarily responsible for cytotoxic activity (17). Since resident CD103+CD8+ T cells are exposed to the endometrial mucosal environment for longer than CD103-CD8+ T cells, this may be a general characteristic of all CD103+CD8+ T cells in the female reproductive tract (17). In contrast, non-resident CD103-CD8+ T cells would be exposed to the endometrial environment for only a short period of time prior to re-entry into systemic circulation, thus minimizing the effect of the local environment on their phenotype. Alternatively, others have reported a spatial separation in the distribution of CD8+T cells in endometrial carcinomas, with CD103- cells primarily located in the tumor stroma and CD103+ cells in the tumor epithelium (11). Thus, the immediate vicinity in which these cell populations are localized, and their exposure to the influence of adjacent cells and secretions, are distinct and may affect their cytotoxic function, as discussed below.

Cell secretions are an important component of the tumor environment. We demonstrated that endometrial carcinoma secretions potently suppress blood CD8+ T cell cytotoxic killing as well as their intracellular content of PRF and GZB, which are essential for mediating the cytotoxic response, to a greater extent than secretions from non-cancerous endometrium. This correlates with the suppressed cytotoxic activity and reduced expression of GZB and PD-1 in CD8+ T cells from endometrial tumors compared to those from non-cancerous tissue. The similarity in intracellular GZB suppression between tumor and blood CD8+ T cells *in vitro* supports our hypothesis that soluble factor(s) released by tumor cells play a critical role in regulating aspects of cytotoxic activity of CD8+ T cells *in situ*. However, the secretions from endometrial carcinomas had no effect on GZA, but did reduce PRF, expression in blood CD8+ T cells. This is in contrast to the CD8+ T cells within the tumor which had reduced expression of GZA, and no change in PRF expression, compared to CD8+ T cells from non-cancerous tissue. The reasons for the differences in the expression of GZA and PRF in our studies may be due to several factors including our use of blood CD8+ T cells which are different from the phenotype of CD8+ T cells at the mucosal surface. Additionally, blood CD8+ T cell were incubated with CM for only 24 hrs prior to analysis. This may not be a sufficiently long exposure time for the CM to fully exert its effect upon the blood CD8+ T cells. A longer incubation interval is likely necessary for CM to affect PRF and GZA expression.

To understand the mechanisms contributing to suppression of CD8+ T cell cytotoxic activity, we determined the composition of secretions from endometrial carcinoma cells in culture. We found that endometrial carcinoma secretions are a complex mixture consisting of multiple cytokines, chemokines, and growth factors, some of which are known to suppress aspects of CD8+ T cell function. Tumor secretions, when compared to secretions from adjacent non-cancerous endometrium, contained significantly higher levels of TGFβ, an immunosuppressive mediator, that we have previously shown directly suppresses CD8+T cell cytotoxicity of both pre- and post-menopausal endometrial CD8+T cells from women without cancer (17). VEGF, another cytokine present at higher levels in tumor CM than adjacent CM, also suppresses multiple aspects of CD8+T cell function including cytotoxicity (30). Alternatively, some soluble mediators present in tumor CM are known to act on intermediary cells that in turn regulate CD8+ T cell function. For example, PDGF-AA enhances the development of Treg cells (31), which in turn may inhibit CD8+ T cell killing. Thus, tumor CM probably exerts its effects on CD8+ T cells at multiple levels, both directly and *via* intermediate cells. A single soluble mediator is unlikely to be wholly responsible for the suppressive effects of tumor CM. Instead, multiple mediators likely act in concert to inhibit CD8+ T cell function and enhance tumor survival.

A previous study (25) also characterized the composition of endometrial carcinoma secretions. Similar to our results they found an increased concentration of IP-10, and no difference in IL-15 and IFNγ, in tumor CM compared to non-cancerous CM. However, in contrast to our results in which IL-1β was lower in tumor CM compared to non-cancerous CM, Degos et al. reported significantly higher levels of IL-1β in tumor CM. One reason for this variation could be differences between our protocols to generate CM. Degos et al. recovered their supernatants during tissue digestion (25) whereas our supernatants were generated after tissue digestion and following a further 24 hrs of incubation. Therefore, soluble mediators in our CM preparations were accumulated over a longer timeframe and after tissue dissociation was complete. The extent to which the secretion profile of endometrial carcinomas varies with tumor stage is also unknown.

The suppression of killing by CD8+ T cells is probably necessary for tumor growth and subsequent metastasis. Beyond their secretions, endometrial tumor cells can suppress the immune response *via* contact-mediated pathways utilizing cell-surface receptors such as PD-L1 to modulate CD8+ T cell function. Previous studies have shown that PD-1/PD-L1 interactions exert a potent immunosuppressive effect on CD8+ T cells, and blockade of PD-1/PD-L1 binding enhances T cell function including cytotoxicity (32). Others have demonstrated that PD-L1 and PD-1 are expressed in endometrial carcinomas to varying degrees (33–41). Endometrial cancers that express high levels of PD-L1 also have increased numbers of cytotoxic CD8+T cells (42). Multiple clinical trials investigating whether blockade of PD-1/PD-L1 signaling leads to improved outcomes in endometrial cancer have reported promising results suggesting the PD-1/PD-L1 signaling has a key role in endometrial tumor progression (43). These past studies provided a rationale for investigating the expression of PD-1 on CD8+T cells. Building upon this solid foundation, we hypothesized that alongside the suppression of cytotoxic killing, PD-1 expression would be elevated on CD8+T cells from endometrial tumors compared to those from adjacent tissue. Unexpectedly we found that PD-1 expression levels were lower on tumor CD8+ T cells than adjacent CD8+ T cells. The reasons for this paradoxical observation are unclear. This suggests that direct interactions between PD-L1, which is known to be expressed on endometrial tumor cells (33), and PD-1 on CD8+ T cells, is unlikely to account for the suppression of killing by CD8+ T cells from endometrial carcinomas. If PD-1/PD-L1 interactions were the primary regulator of cytotoxic killing, we would expect PD-1 levels to be higher on tumor CD8+ T cells than adjacent CD8+ T cells. Our paradoxical results could be explained by the restriction of our study to early Stage I and II endometrial carcinomas. This is in contrast to clinical trials which focus on advanced stage or metastasized endometrial carcinomas (43). Our findings indicate the importance and need for further studies to investigate the importance of the PD-1/PD-L1 axis suppression of cytotoxic killing by CD8+ T cells in advanced stage endometrial cancers.

One explanation for the concurrent reduction in cytotoxic killing and expression of PD-1 on tumor CD8+T cells could be that beyond the PD-1/PD-L1 axis other checkpoint inhibitors may inhibit CD8+ T cell cytotoxicity (44) in Stage I endometrial tumors. For example, the V-domain Ig suppressor of T cell activation (VISTA) (45), suppresses CD8+ T cell proliferation, cytokine production, and the number of tumor infiltrating CD8+ T cells (46). In addition, the checkpoint inhibitor CTLA-4 prevents the activation of cytotoxic T cells (47). CTLA-4 is expressed in multiple gynecologic cancers (48, 49) and clinical data shows that CTLA-4 blockade may enhance patient outcomes in specific endometrial cancers (50). Expression of Tim-3, another checkpoint inhibitor, is linked to an exhausted phenotype of CD8+ T cells and is a negative regulator of CD8+T cytotoxicity (51). Tim-3 is expressed in endometrial cancers (52) and is present on endometrial tumor NK cells suggesting these cells may have an exhausted phenotype (25). TIGIT, another marker of exhaustion (53), is also expressed by endometrial tumor NK cells (25). Whether VISTA, CTLA-4, TIM-3, and other immune regulators suppresses CD8+ T cell cytotoxic killing in our system is unknown but represents a promising avenue for future research that could explain why PD-1/PD-L1 interactions do not suppress tumor CD8+ T cell killing in our system.

Distant from the tumor, changes in the peripheral environment may affect CD8+ T cell function. Serum estradiol (E_2_) levels are higher in endometrial cancer patients compared to healthy controls (54) and are linked to greater risk of endometrial cancer in post-menopausal women (55, 56), partly *via* the proliferative effects of unopposed E_2_ on the endometrium. While post-menopausal women do not secrete ovarian E_2_, adipose tissue still produces E_2_ which enters systemic circulation. Increased obesity, and thus adipose tissue, is also linked to increased risk of endometrial cancer (57). Therefore, elevated levels of E_2_ in endometrial cancer patients may act directly upon CD8+ T cells and inhibit their cytotoxic capacity. Recently we have shown that E_2_ is a potent suppressor of endometrial CD8+ T cell cytotoxic activity (23), and that CD8+ T cell cytotoxic activity undergoes cyclical changes in the normal pre-menopausal endometrium, with maximal suppression during the secretory phase (16, 17). Furthermore, E_2_ treatment leads to a reduction in intracellular levels of GZA in endometrial CD103+ and CD103- CD8+ T cells (23). In our studies, we found decreased levels of GZA in tumor CD103-CD8+ T cells but not CD103+ cells (**Figure 4**). Since CD103-CD8+ T cells are not resident in the endometrial mucosa, their exposure to systemic E_2_ may lead to suppressed GZA in endometrial carcinoma patients. Overall, the effect of E_2_ on immune cell function in endometrial carcinoma remains relatively unknown and requires further study.

In conclusion, CD8+ T cell cytotoxicity in endometrial carcinomas is markedly suppressed relative to those found in adjacent non-tumor endometrial tissues. Suppression of cytotoxic killing by CD8+ T cells correlates with the reduction of intracellular cytolytic molecules that is in part regulated by soluble factors produced and secreted into tumor microenvironment. These studies demonstrate the complexity of CD8+ T cell regulation within endometroid tumors and provide a foundation for future studies to investigate the endometrial tumor microenvironment and its modulation of immune function.

## Data Availability Statement

The raw data supporting the conclusions of this article will be made available by the authors, without undue reservation.

## Ethics Statement

The studies involving human participants were reviewed and approved by Dartmouth College Institutional Review Board and the Committee for the Protection of Human Subjects (CPHS). The patients/participants provided their written informed consent to participate in this study.

## Author Contributions

MP and ZS performed the experiments. LT oversaw recovery of tissue following surgery. MP, ZS, MR-G, LT, EU, and CW all contributed to the design of the experiments and writing of the manuscript. All authors contributed to the article and approved the submitted version.

## Funding

Research reported in this publication was supported by NIH grants AI117739 (CRW), AG064794 (CRW), and a Prouty Pilot Grant from Friends of the Norris Cotton Cancer Center (CRW). Shared resources of the Immune Monitoring and Flow Cytometry Resource at the Norris Cotton Cancer Center at Dartmouth were from NCI Cancer Center Support Grant (P30 CA023108) and COBRE Grant (P30GM103415-15) from the National Institute of General Medical Sciences.

## Conflict of Interest

The authors declare that the research was conducted in the absence of any commercial or financial relationships that could be construed as a potential conflict of interest.
